# Decoding the cellular landscape of biological stress in the human brain

**DOI:** 10.1016/j.ynstr.2026.100782

**Published:** 2026-01-22

**Authors:** Natalie Matosin

**Affiliations:** a The University of Sydney, School of Medical Sciences, Faculty of Medicine and Health, Australia; b The University of Sydney, Charles Perkins Centre, Australia; c The University of Sydney, Brain and Mind Centre, Australia

## Abstract

Adversity exposure leading to a dysfunctional biological stress response represents a significant risk factor underlying psychiatric disorder aetiology for many individuals. Yet our understanding of how different cell types within stress-responsive brain circuits differentially contribute to psychiatric risk remains limited, particularly in the human brain. Our lab, the Mental Illness, Neurobiology and Disorders of Stress (MINDS) Laboratory, has been addressing this knowledge gap through large-scale analyses of postmortem human brain specimens from individuals with major psychiatric disorders who experienced high levels of adversity in their lives. We have a focus on examining targets and pathways involved in HPA axis and glucocorticoid-mediated signalling in the human brain at single-cell resolution. Through integration of single-cell and spatial molecular data with advanced histological approaches to evaluate cellular morphology, we have identified cell-type-specific vulnerability patterns to the biological consequences of adversity exposure. Our findings demonstrate how different populations respond to adversity through coordinated molecular and morphological changes that affect synaptic function and stability. This approach exemplifies the potential for combining new spatial omics and traditional histological approaches to achieve precision medicine in psychiatry, by revealing specific cellular targets for therapeutic intervention. Our work facilitates a shift from broad neurotransmitter-based interventions towards targeted therapeutic strategies for stress-related psychiatric disorders. These advances provide a foundation for developing more effective treatments tailored to the underlying cellular pathology in individual patients with stress-related mental illness.

## 1 Introduction

Adversity exposure that leads to a prolonged biological stress response with lasting effects on an individual's functioning is among the strongest risk factors for psychiatric disorders (Cohen et al., 2007; Kessler et al., 2010; Schneiderman et al., 2005). To study the underlying mechanisms of how this occurs, clearly defining ‘stress’, ‘adversity’ and ‘biological stress’ is important. In our lab, we refer to ‘stress’ overall as it is used in colloquial language, representing the broad construct of a state of worry or mental tension due to a difficult situation. In more discipline-specific terms, we further conceptualise adversity as a rainstorm; and biological stress as getting wet: adversity represents the external environmental conditions (abuse, neglect, poverty) while biological stress encompasses the biological responses triggered by these exposures within the organism (Remmers et al., 2024). For decades, our understanding of biological stress has been conceptualised through hypothalamic-pituitary-adrenal (HPA) axis theory (e.g. McEwen, 1992; Yehuda et al., 1993). This perspective provides crucial insights into how impaired glucocorticoid-mediated stress responsivity contributes to the aetiology of psychiatric disorders (Heim et al., 2008; Kitraki et al., 1999; McEwen, 1992; Yehuda et al., 1993).

While animal studies have been instrumental in demonstrating how dysregulation of the HPA-axis can recapitulate behavioural, cellular and molecular phenotypes characteristic of stress-related psychiatric disorders, no single stress model can fully recapitulate the inherent complexity of the human biological stress response following adversity exposure (Tran and Gellner, 2023). This is significant given magnetic resonance imaging studies of the human brain consistently reveal that exposure to high levels of adversity (e.g., abuse, neglect, poverty) are associated with system-level alterations and macrostructural changes in a region-dependent manner based on the specific stressor experienced (e.g. Heim et al., 2013; Tomoda et al., 2012). This systems-level understanding has helped inform molecular approaches, which have then consolidated evidence that HPA axis stress responsivity following exposure to adversity is a significant risk factor underlying the pathogenesis of psychiatric disorders (Gillespie et al., 2009; Heim et al., 2008). This occurs through alteration of neurotransmitter systems, gene expression patterns, and protein signalling cascades which govern changes in cytoarchitecture and ultimately, brain circuitry (Kaul et al., 2021; Stephens and Wand, 2012). However, despite decades of research, we still lack a comprehensive understanding of how different cell types within stress-responsive brain circuits contribute to heterogeneity in stress responsivity and psychiatric risk.

We now have the means to rapidly and systematically sift through cell-type specific cellular, anatomical and molecular features within brain areas involved in processing stress and psychopathology. Advances in single-cell and spatial omics technologies have provided unprecedented tools to systematically examine brain cytoarchitecture and molecular composition at high resolution (Curry et al., 2024; Edmond and Matosin, 2025). These approaches reveal substantial heterogeneity in how different brain cell populations respond to environmental stress signals (Curry et al., 2024; Edmond and Matosin, 2025; Miranda et al., 2023). Unsurprisingly, these emerging layers of evidence have shifted how we conceptualise the neurobiology of stress in the context of psychiatric illness, highlighting that the brain consists of diverse cell populations with distinct molecular profiles and stress sensitivities (e.g. Cruceanu et al., 2022; Lopez et al., 2021). This is providing a new level of understanding of pathological processes and facilitating precise identification of targets for therapeutic intervention.

**Fig. 1:**
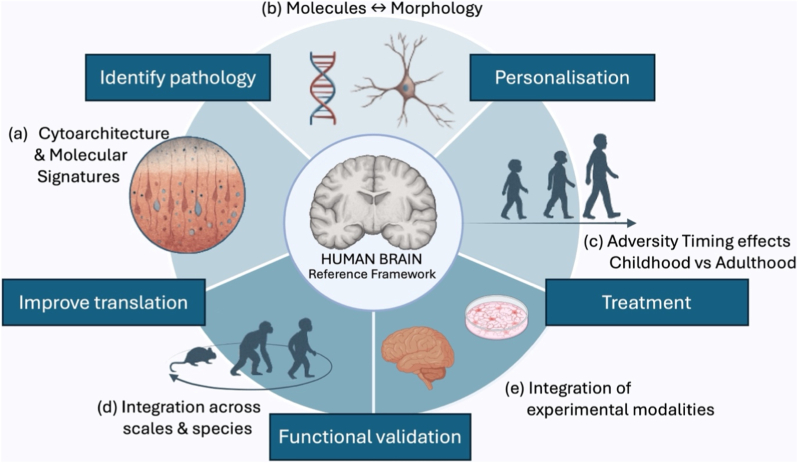
The MINDS Lab investigates the fine-grained neuropathology of the human brain to advance disease modelling and enable personalised medicine approaches in psychiatry. Our research program centres on: (a) comprehensive mapping of human brain cytoarchitecture and molecular composition, (b) defining the relationship between molecular signatures (genome, epigenome, transcriptome, proteome, metabolome) and cellular morphology, and (c) understanding how adversity and its timing shape brain structure and function. By integrating data across (d) scales, species, and (e) experimental modalities, our vision is to build a detailed reference framework of the healthy human brain. This framework will allow us to identify pathological changes contributing to the onset and progression of psychiatric disorders, enable functional validation and improve translation, define biologically meaningful subgroups of individuals with shared disease mechanisms, and pinpoint therapeutic targets to guide tailored treatment strategies.

In the Mental Illness, Neurobiology and Disorders of Stress (MINDS) Lab, we believe that elucidating the persistent, cell-type specific consequences of high-adversity exposure in the human brain is key to understanding why some individuals develop psychiatric illnesses while others show remarkable resilience to similar environmental challenges, and to identifying new pathways for precise and effective pharmacological treatment. Building on our research program summarised in Fig. 1, the MINDS Lab will continue to map human brain cytoarchitecture and molecular composition, define how multi-omic signatures relate to cellular morphology, and determine how the timing and nature of adversity shape brain structure and function. By integrating these fine-grained data across scales, species, and experimental modalities into a detailed reference framework of the healthy and diseased human brain, future work will identify pathological changes that drive psychiatric disorders, refine biologically meaningful subgroups with shared disease mechanisms, and pinpoint therapeutic targets that can guide personalised treatment strategies in psychiatry – an international research priority in the field of psychiatry (Stephan et al., 2016).

## 2 The cellular mosaic of stress vulnerability

A large part of current knowledge about how adversity affects the human brain comes from magnetic resonance imaging studies. For example, adversity exposure has been associated with reduced hippocampal volume, altered prefrontal cortex function, and changes in amygdala regulation (summarised in Table 1 Kaul et al., 2021). However, the cellular and molecular mechanisms that contribute to these macrostructural findings remain incompletely understood. It is not yet clear whether such volumetric alterations reflect changes in dendritic spines, branching, neuropil, or shifts in cell populations, sizes, or numbers. Ongoing work in our lab seeks to address these questions within stress-sensitive regions of the human brain.

A region that we have focused on intensely is the orbitofrontal cortex, which is a critical integration point involved in modulating how the human brain responds to adversity. Non-human primate tract-tracing work has shown that the orbitofrontal cortex receives afferent input from the subgenual anterior cingulate cortex and integrates signals from the limbic cortex, amygdala, and mediodorsal thalamus (Barbas, 2007), patterns that closely parallel those described in human neuroanatomical studies (Heather Hsu et al., 2020). The orbitofrontal cortex also plays an efferent role, sending outputs to the striatum, brainstem, and hypothalamus, thereby influencing physiological systems such as heart rate and cortisol release (Barbas, 2007) which are directly relevant for understanding stress-responsive circuitry in humans. The orbitofrontal cortex is therefore a key functional hub in the brain, essential for emotional learning and the integration of emotional experiences with visceromotor responses (Rolls and Grabenhorst, 2008). On a macrostructural scale, there are several reports of reduced orbitofrontal cortex volume in individuals exposed to severe forms of early life adversity (Gold et al., 2016; Hanson et al., 2010; Holz et al., 2015; Monninger et al., 2020), suggesting this is a region particularly vulnerable to a heightened biological response.

In Kaul et al. (2020), we examined the lasting impact of adversity exposure on neuronal morphology in the orbitofrontal cortex. Using medical records from the NSW Brain Tissue Resource Centre in Sydney, Australia, we identified brain donors with a major psychiatric diagnosis (depression, bipolar disorder or schizophrenia spectrum disorders) who had experienced high levels of adversity either early or later in life (Kaul et al., 2020). High adversity exposure was defined as an event or series of events that involved emotional and physical harm or threat, with a lasting impact on the individual's functioning or their emotional, social and physical wellbeing. This information was gathered from extensive clinical records, where the adversity was determined to be severe (e.g. physical or sexual trauma, abuse, childhood neglect) or a clear trigger in the emergence of psychopathology (e.g. complex divorce, loss of a child, a major accident). We then compared these cases to psychiatric and control cases with relatively low adversity exposure in the medical records. This adversity stratification framework enabled my lab to curate a novel postmortem cohort that, for the first time, accounted for the timing of adversity exposure (early vs later in life). The time at which adversity occurs is a critical factor, given evidence that early life and adult adversity have distinct biological consequences (Friedman et al., 2015). Importantly, a trans-diagnostic approach examining shared mechanisms across multiple psychiatric disorders rather than focusing on individual diagnostic categories, is also essential as mounting evidence suggests that adversity is the leading environmental risk factor shared across multiple psychiatric disorders (Hostinar et al., 2023). This transdiagnostic perspective is supported by growing recognition of genetic overlap and shared symptomatology across psychiatric conditions (Stephan et al., 2016), and continues to inform the approach we take as a lab with multi-omic datasets in other brain areas and cohorts. Importantly, while the biological stress response following adversity exposure involves HPA-axis activation that can be measured through constituents such as glucocorticoid levels (Dickerson and Kemeny, 2004), postmortem human brain tissues provide only a static snapshot at the time of death. Thus, what we are measuring in our studies is the lasting impacts of exposure to adversity, that is, the environmental conditions that would elicit a biological stress response in living individuals.

Within this newly curated postmortem cohort, we applied Golgi-Cox staining to brain sections and quantified the morphology and density of over 22,000 dendritic spines on layer-specific pyramidal neuron apical dendrites in the human orbitofrontal cortex (Kaul et al., 2020). Our findings revealed that high adversity exposure in both childhood and adulthood was associated with substantial reductions in mature mushroom spine density. Strikingly, we found these losses could amount up to 56 % in both the superficial (layers II/III) and deeper (layer V) cortical layers. When comparing the outcomes of childhood and adulthood adversity exposure, we found that high adversity in childhood was associated with significantly greater reductions in both total and mature mushroom spine density across both cortical layers. In the superficial layers (II/III), childhood adversity was associated with 52–56 % reductions in mushroom spine density (P < 0.01), while adulthood adversity showed only a non-significant trend (−30 %, P = 0.059). Similarly, in the deeper layer V, childhood adversity was associated with 53 % reductions in mushroom spine density (P = 0.019), with less pronounced effects observed for adulthood adversity. This work highlighted the timing of adversity exposure is a key determinant of neuronal vulnerability to stress in the orbitofrontal cortex, and suggests that altered neuronal morphology may be at least partially responsible for reduced orbitofrontal cortex volume in response to high adversity exposure (Kaul et al., 2020).

## 3 From morphology to molecules and back again

Our morphological profiling of the orbitofrontal cortex laid the groundwork for our subsequent molecular studies using adversity stratification frameworks. Beyond neuronal populations, we have identified that glial cells, particularly astrocytes, are also vulnerable to biological stress following adversity, contributing to prolonged and dysregulated stress responses that can disrupt neural circuit function and increase risk for psychiatric illness (Kaul et al., 2025). Astrocytes are the brain's most abundant cell type and serve as essential regulators of synaptic function, managing neurotransmitter clearance, metabolic support, and the balance between excitatory and inhibitory signalling (Kaul et al. 2022). Astrocytes are strategically positioned at neuronal synapses and as indivisible components of the blood brain barrier (Gradisnik and Velnar, 2023). Together, this makes astrocytes prime responders to circulating stress hormones like cortisol and implicates them as key intermediaries of the biological stress response via glucocorticoid-mediated stress responsivity and neural circuit dysfunction (Heard et al., 2021; Tertil et al., 2018).

Through comprehensive profiling of over 145,000 human cortical astrocytes using single-nucleus (Frohlich et al., 2024) and spatial transcriptomics Kaul et al. (2025), our recent work has found that astrocytes in the orbitofrontal cortex comprise a molecularly and anatomically diverse population organised into distinct functional clusters (Kaul et al., 2025). Each cluster showed enrichment for specific biological functions: some specialised in synaptic regulation and neurotransmitter transport, others in metabolic homeostasis or some immune-related signatures. By integrating spatial transcriptomics, we were also surprised to discover that the anatomical location of the astrocytes was encoded in the RNA, with most of our delineated astrocyte clusters showing anatomical specificity to either the cortical grey- or white-matter (Kaul et al., 2025).

Interestingly, when examining individuals with major psychiatric disorders (depression, bipolar disorder or schizophrenia) who had experienced high-adversity exposure, we found that only one specific astrocyte population, cluster 2, showed significant alterations (Kaul et al., 2025). Cluster 2 astrocytes were uniquely enriched for glutamate-related synaptic functions, specifically reduced expression of the glutamate transporter EAAT2 and impaired glutamate-glutamine cycling. In contrast, neighbouring astrocyte populations within the same tissue samples remained unaffected, maintaining typical molecular signatures despite similar adversity histories. This pattern suggests that adversity is not associated with widespread astrocyte dysfunction but that it localises in subpopulations with key roles in excitatory neurotransmission.

Given that glutamate is the brain's primary excitatory neurotransmitter, astrocyte-mediated glutamate clearance is therefore essential for preventing excitotoxicity and supporting synaptic function (Andersen et al., 2021). Thus, alteration in this specific astrocyte subtype may have broader consequences for neural circuit integrity (Kaul et al., 2025).

## 4 The critical importance of adversity timing on the human brain

Our astrocyte findings highlight that the timing of adversity exposure is associated with the extent and nature of cellular alterations in individuals that lived with psychiatric disorders. When we stratified our cohort based on the timing of first severe adversity exposure (childhood <12 years, adolescence 12–18 years, early adulthood 18–25 years, adulthood >25 years), we found that earlier adversity exposure was associated with more pronounced transcriptomic and morphological changes in astrocytes (Kaul et al., 2025). High childhood adversity exposure was associated with the most alterations in astrocyte gene expression patterns, with stronger effect sizes and more widespread transcriptomic changes compared to adversity experienced later in life. This timing effect was evident not only in gene expression data but also in morphological analyses, where an increase in EAAT2-positive astrocyte density and cellular localisation was most pronounced in individuals with a history of childhood adversity (Kaul et al., 2025).

These findings align with broader literature demonstrating that early life represents a critical period for brain development, during which environmental stimuli can have lasting effects on cellular architecture and function (Friedman et al., 2015; Heim et al., 2010). The heightened vulnerability of developing brain cells to high adversity exposure suggests that early intervention strategies may be particularly important for preventing long-term cellular dysfunction and associated psychiatric risk (Graham et al., 2021). The timing of adversity exposure therefore appears to be critical, with childhood adversity associated with more pronounced cellular and molecular changes than later-life adversity (Albott et al., 2018). This supports clinical evidence linking early trauma to heightened psychiatric risk and highlights the potential for early detection and targeted interventions aimed at preserving cellular function in the vulnerable developing brain (Graham et al., 2021).

## 5 The cell-type specific signatures of biological stress in psychiatric disorders

**Fig. 2:**
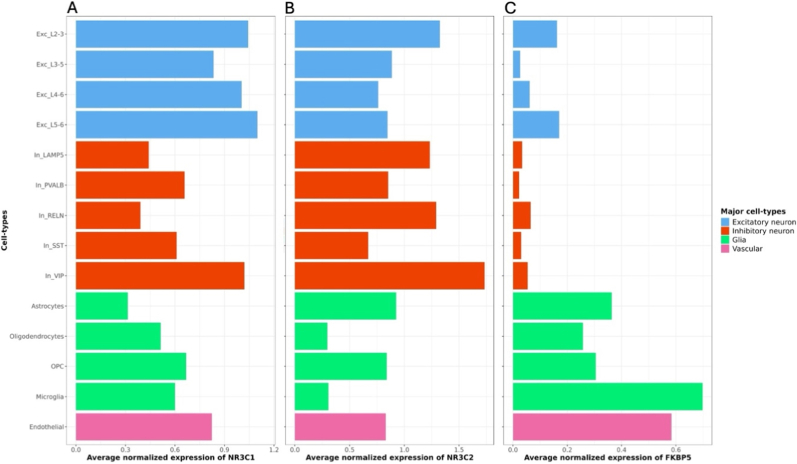
Example of the cell-type specific expression patterns of genes related to HPA axis signalling (a) the glucocorticoid receptor *NR3C1*, (b) the mineralocorticoid receptor *NR3C2*, and (c) the glucocorticoid co-chaperone *FKBP5* in the orbitofrontal cortex of postmortem human brain. Data derived from Frohlich et al. (2024), where donor details have been previously published.

To understand how early adversity exposures leave a biological imprint, we must examine how molecular stress systems, such as glucocorticoid signalling, interact with the brain's cellular landscape, particularly the distinct molecular signatures that shape the vulnerability or resilience of each cell population to environmental challenge (McEwen et al., 2015). The human brain contains an extraordinary diversity of cell types, with recent studies estimating over 3000 distinct cellular populations across regions (Maroso, 2023; Nusinovich, 2024). Each population is defined by its morphology, connectivity, and unique profiles of stress-responsive molecular machinery (Siletti et al., 2023). For example, the presence or absence of cortisol receptors (Fig. 2a–b), the expression patterns of stress-responsive genes like *FKBP5* (Fig. 2c), and the cellular capacity to respond to neurotransmitters, neuroactive steroids and neuropeptides (Edmond, 2025) all contribute to a cell's vulnerability or resilience to stress exposure. These features converge at the circuit and systems level, producing cell- and context-specific responses dependent upon the cellular and molecular milieu of the brain region involved. These patterns of expression may also be dynamic across development and the human life course (Cruceanu et al., 2022; Dony et al., 2025; Matosin et al., 2023).

## 6 *FKBP5* in cortical cell type-specific stress vulnerability

Building on these cell type-specific expression patterns of HPA axis genes, *FKBP5* provides an example of how stress signalling pathways become selectively dysregulated in defined cortical neuronal populations, creating patterns of cellular vulnerability over the lifespan that can be mapped across multiple levels of molecular analysis (Binder, 2009; Matosin et al., 2018; Zannas et al., 2016). *FKBP5* encodes FK506-binding protein 51 (FKBP51), an allosteric heat shock protein 90 (HSP90) co-chaperone of the glucocorticoid receptor that is highly responsive to glucocorticoid-mediated stress signalling (Binder, 2009). What makes *FKBP5* particularly important is its role as both a propagator and terminator of the HPA axis regulated biological stress response (Zannas et al., 2016). Although it is essential for physiological stress signalling, *FKBP5* contributes to pathology when its expression exceeds normal levels (Matosin et al., 2018).

Building on a series of landmark studies by Dr. Elisabeth Binder (Binder, 2009; Binder et al., 2004, 2008), we performed a large-scale postmortem study examining over 1000 individuals across six cohorts (Matosin et al., 2023). Our work demonstrated there are specific patterns of *FKBP5* dysregulation in schizophrenia spectrum disorders, major depression and bipolar disorder. Specifically, expression of *FKBP5* mRNA naturally increases with age in the neurotypical brain (Matosin et al., 2018, 2023; Weickert et al., 2015). In psychiatric cases, particularly individuals with schizophrenia, *FKBP5* demonstrates a significantly heightened gene expression (Matosin et al., 2023). With single-nucleus RNA sequencing across multiple regions of the cortex, such as the dorsolateral prefrontal cortex, orbitofrontal cortex and anterior cingulate cortex, we localised these alterations, reporting that pathological elevation of *FKBP5* occurs predominantly in excitatory superficial layer neurons of the neocortex (Matosin et al., 2023).

This cellular specificity has implications that extend beyond simple gene expression patterns. In these same superficial layer cortical neurons showing elevated *FKBP5*, strong inverse correlations with *BDNF* expression and strong reductions in dendritic spine density were observed (Matosin et al., 2023). The integration of molecular and morphological data suggests that neurons with the highest *FKBP5* expression have up to ∼50 % loss of mature mushroom spines, which represent the most stable form of excitatory synapse (Matosin et al., 2023). Identification of these specific molecular changes, which co-occur with selective alterations in the morphology of superficial layer neurons, has implications for circuit function, as mushroom spines are characterised by large spine head volumes that correlate with synaptic strength and stability (Bourne and Harris, 2008; Hotulainen and Hoogenraad, 2010; Kaul et al., 2021). This selective vulnerability of cortical mushroom spines suggests that biological stress impacts the most functionally important synaptic connections whilst sparing less mature spine types. These findings raise important questions about how such cell-type-specific molecular and morphological changes could be leveraged for more targeted therapeutic interventions.

The cell-type specificity of *FKBP5* dysregulation has important implications for therapeutic development. Traditional approaches in psychiatry have primarily focused on targeting neurotransmitter systems through broad receptor-based interventions. In contrast, our findings exemplify how we can narrow down on more specific cellular targets, in this case, superficial layer excitatory neurons of the cortex with elevated *FKBP5* expression and altered synaptic architecture (Matosin et al., 2023). While direct, cell-type specific targeting of FKBP51 requires feasibility testing and development, future strategies could focus on identifying genes that are co-regulated with *FKBP5* and more selectively expressed in vulnerable cell populations and/or subpopulations (Van de Sande et al., 2023). Additionally, our most recent work demonstrates that cortical *FKBP5* DNA methylation patterns in the proximal enhancer are altered in psychiatric disorders and negatively correlate with gene expression, particularly in schizophrenia, providing potential epigenetic targets to direct or localise treatment effects (Edmond et al., 2025). The identification of CpG-specific methylation changes associated with glucocorticoid response elements in the human cortex might indicate that epigenetic interventions could offer a mechanism-based approach to modulate *FKBP5* expression in specific patient subgroups. Such markers may offer more precise avenues for intervention, either through cell-specific delivery of therapeutic agents or as alternative therapeutic targets (Dai et al., 2024).

Our body of work centred on *FKBP5* demonstrates how cell type-specific dysfunction in psychiatric disorders is not solely driven by isolated changes in gene expression. Our findings suggest that pathological features of psychiatric disorders may arise through coordinated disruptions in broader transcriptional networks in the human cortex (and possibly more broadly across the brain) at the levels of the genome, epigenome, transcriptome, and proteome (Edmond et al., 2025; Matosin et al., 2023). Single-cell analyses reveal that these changes are highly structured, associated with specific cell populations in a reproducible manner (Edmond et al., 2025; Matosin et al., 2023). These studies begin to provide a step towards uncovering biological stress processes at a high resolution to facilitate the identification of feasible, precise and druggable targets.

Psychiatric disorders encompass diverse cellular and molecular pathologies that may manifest as similar symptom phenotypes (Hostinar et al., 2023). For instance, patients with major depression may have distinct cellular dysfunctions requiring different treatments, which partly explains the limited success of uniform treatment approaches. Stratifying patients by both biological markers and adversity exposure profiles rather than symptoms alone could improve clinical trial design and treatment selection (Stephan et al., 2016). By grouping individuals based on molecular signatures of cellular dysfunction identified in postmortem brain studies (such as *FKBP5*) clinical research can better target subpopulations likely to respond to specific therapies. Thus, a cellular-level understanding of stress vulnerability holds significant promise for advancing precision psychiatry.

## 7 Integrating scales and species

Understanding vulnerability to a dysregulated stress response across psychiatric disorder phenotypes requires a multi-faceted and integrative approach. While human postmortem brain tissue provides valuable insight, it only offers a static snapshot in time and lacks experimental control (Harrison, 2011; Lewis, 2002; McCullumsmith et al., 2013). These limitations can be addressed by combining complementary methodologies, including comparative studies across species to identify evolutionarily conserved cellular and molecular biological stress mechanisms (e.g. Lindhout et al., 2024; Zhou et al., 2025). *In vitro* modelling is also highly valuable for modelling functional aspects of a cellular system or cell type, such as we have achieved for glucocorticoid signalling in astrocytes (e.g. Kaul et al., 2025). Such comparative perspectives are crucial for prioritising which mechanisms represent vulnerabilities to a heightened or persistent biological stress response versus those arising from human-specific features such as extended cortical development and increased neuronal complexity. Functional validation in model systems informed by this conservation can enhance translational relevance.

Moving beyond the traditionally studied brain regions such as the prefrontal cortex and hippocampus will also be essential to systematically profile biological stress responses throughout the brain. As single-cell technologies continue to evolve, combining multiple levels of regulatory processes across the central dogma – from gene to protein and all the way to cell morphology and circuitry – can reveal broader mechanisms of upstream and downstream gene expression regulation that drives cellular responses to biological responses to stress. This multi-scale approach, which is a defining feature of our lab's work, aims to map the full regulatory cascade underlying cell-type specific vulnerability, from upstream signalling to transcriptomic changes and structural alterations, highlighting multiple points for potential therapeutic intervention (Curry et al., 2024; Edmond and Matosin, 2025; Kaul et al., 2021).

Advances in computational methods, including digital pathology and artificial intelligence, will be vital to integrate diverse data types across species and experimental platforms (Song et al., 2023; Zarella et al., 2023). These integrative models hold promise for predicting cellular vulnerability and guiding therapeutic development (Son et al., 2024). Ultimately, a convergence of evolutionary biology, experimental neuroscience, and computational analysis will be necessary to develop precise, biologically informed interventions tailored to the cellular and molecular mechanisms underlying psychiatric disorders (O'Connor et al., 2023).

## 8 Conclusions and future directions

Advances in single-cell and spatial omics technologies are revolutionising our understanding of the human brain and cell-types specific dysfunction in psychiatric disorders. Our detailed analysis of the orbitofrontal cortex illustrates how combining these approaches with traditional histology can reveal specific cellular changes linked to adversity that may reveal highly precise treatment targets (Frohlich et al., 2024; Gerstner et al., 2025; Kaul et al., 2020, 2025; Matosin et al., 2023). Moving forward, key priorities include bridging postmortem findings with functional insights through *in vivo* and *in vitro* models, cross-species integrations, and developing peripheral and neuroimaging biomarkers that reflect cellular dysfunction within the brain to facilitate early detection of those at risk for developing a psychiatric disorder. Moreover, it will be important to move beyond commonly studied regions, such as the dorsolateral prefrontal cortex, in postmortem studies to systematically profile cell-type specific stress responses across the whole brain to improve our understanding of interregional interactions driving neural circuit dysfunction and dysregulated biological responses to adversity. Integrating multi-omics data including spatial epigenomics, transcriptomics, and proteomics with new computational tools will be critical to uncover the pathways through which adversity embeds biological risk for psychiatric disorders (Curry et al., 2024; Edmond and Matosin, 2025). This cellular- and molecular level insight marks a shift from symptom-based treatment towards precision psychiatry, targeting the molecular and cellular origins of mental illness. Considering the nearly one billion people affected globally (Casella et al., 2025), advancing biologically precise interventions is crucial.

The MINDS Lab continues to actively pursue improving our knowledge of the neurobiology of stress by systematically applying adversity stratification frameworks and multi-omic profiling across multiple brain regions and cohorts. We are driven by a desire to identify convergent cellular mechanisms that can be translated into targeted therapeutic interventions with specific molecular-morphological signatures of stress vulnerability, to improve the quality of life for those managing the challenges of living with major psychiatric disorders.

## Declaration of competing interest

none to disclose.

## Data Availability

No data was used for the research described in the article.
